# Anomalous Arising of Right Coronary Artery from the Pulmonary Artery

**DOI:** 10.3390/jcdd11020050

**Published:** 2024-02-01

**Authors:** Adrián Kolesár, Tomáš Toporcer, Jana Čobejová, Štefan Lukačin

**Affiliations:** Department of Heart Surgery, East Slovak Institute of Cardiovascular Disease and Medical Faculty, University of Pavol Jozef Šafárik in Košice, 04011 Košice, Slovakia; akolesar@vusch.sk (A.K.); jcobejova@vusch.sk (J.Č.); slukacin@vusch.sk (Š.L.)

**Keywords:** coronary artery anomalies, ARCAPA, aortic valve stenosis

## Abstract

Coronary artery anomalies are seen in less than 1% of the general population and in 1.6% of cardiac catheterization cases. The anomalous origin of the coronary artery from the pulmonary artery is one of four groups of coronary artery origin anomalies. The incidence of anomalous origin of the right coronary artery from the pulmonary artery is 1 in 500,000 and was first described in 1882 by John Brook. This case report reports on a 67-year-old man with a diagnosis of asymptomatic anomalous origin of the right coronary artery from the pulmonary artery. The patient underwent surgery of the aortic valve because of valve stenosis. A concomitant surgical procedure included repositioning of the right coronary artery origin to the aortic root sinus. The patient was discharged on the 12th postoperative day, in good condition. Anomalous origin of the right coronary artery from the pulmonary artery is commonly asymptomatic, and surgery is required only if myocardial ischemia is presented.

## 1. Introduction

Coronary artery origin anomalies are rare congenital heart diseases (CHD) that are divided into four groups: anomalous origin of the coronary artery from the pulmonary artery (PA); the ectopic origin of the coronary artery from the aortic sinus; the absence of a coronary artery; and a coronary artery fistula [1]. Coronary artery anomalies are seen in less than 1% of the general population and in 1.6% of cardiac catheterization cases [1,2]. Coronary artery origin from the PA is a part of this group, with an incidence of 0.01% in the general population [3,4].

The arising of the left common coronary artery (LCCA) from the PA (ALCAPA), known as Bland–White–Garland syndrome, was first described in 1933 [5,6,7]. This disease represents 0.24–0.46% of all congenital heart malformations, which represents an incidence of 1 in 300,000 [1,4,8,9,10]. ALCAPA is predominantly encountered as an isolated anomaly [7]. Less often, it can be associated with atrial septal defect (ASD), ventricular septal defect (VSD), patent ductus arteriosus, aortic coarctation, aortopulmonary window, tetralogy of Fallot, and a bicuspid aortic valve [6,9]. The independent arising of the left anterior descending coronary artery (LAD) from the PA has also been described [11].

The incidence of anomalous origin of the right coronary artery (RCA) from the pulmonary artery (PA) (ARCAPA) is 1 in 500,000 and was first described in 1882 by John Brook [1]. It represents 0.12% of all congenital heart diseases [1,2,3,4,5,6,7,8,9,10,11,12,13,14]. The lower incidence of ARCAPA in comparison with ALCAPA is caused by the higher intimacy of the left common coronary artery (LCCA) origin to the PA sinus [6]. ARCAPA is in 40% of cases associated with other congenital heart diseases (CHD), including tetralogy of Fallot, patent ductus arteriosus, aberrant right subclavian artery, bicuspid aortic valve, ventricular septal defect (VSD), atrial septal defect (ASD), double outlet right ventricle, coarctation of the aorta, aortic arch hypoplasia, pulmonary stenosis, aortic stenosis, and aortopulmonary window [1,2].

## 2. Case Report

**Figure 1:**
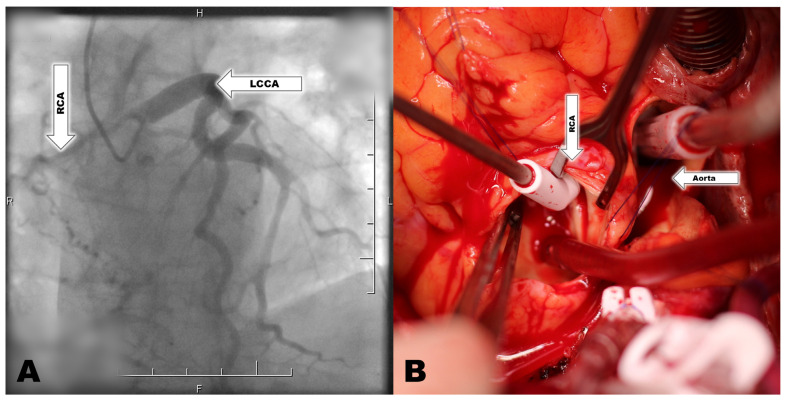
(**A**) Coronarography of the patient with ARCAPA showing a dilated LCCA and retrograde filling of the RCA (F—foot; H—head; L—left side; LCCA—left common coronary artery; R—right side; RCA—right coronary artery); (**B**) Preoperative picture showing application of cardioplegic solution into the orifice of the RCA in the PA (RCA—right coronary artery).

**Figure 2:**
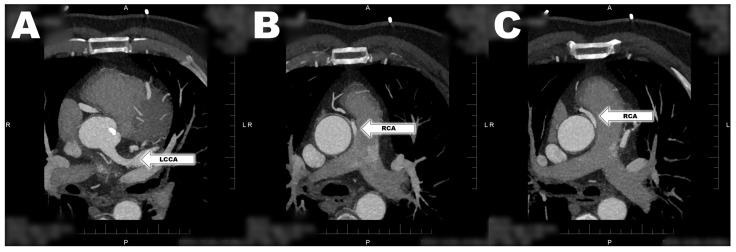
Computed tomography of a patient with ARCAPA. (**A**) View of the dilated LCCA. (**B**) View of the pathological distance of the RCA from the pulmonary artery. (**C**) View of the course of the RCA between the pulmonary artery and the aorta. (A—anterior; L—left side; LCCA—left common coronary artery; P—posterior; R—right side; RCA—right coronary artery).

A 67-year-old man with history of arterial hypertension, dyspnea, diabetes and dyslipidemia was admitted to the department of cardiology because of suspicion of aortic valve stenosis. The patient’s history showed asymptomatic ARCAPA diagnosed five years ago, and mild aortic stenosis diagnosed two years ago. In the past, the patient did not show any signs of ischemic heart disease. The patient was admitted for recent progression of dyspnea on exertion. Angina pectoris and syncope were not recorded in the patient. Echocardiography (ECHO) showed aortic valve stenosis with an aortic valve area of 0.8 cm^2^, peak gradient on the aortic valve of 65 mmHg, median gradient on the aortic valve of 35 mmHg, peak velocity on the aortic valve of 4 m/s, aortic valve regurgitation of grade 2, left ventricle ejection fraction (LVEF) at 65%, and mild mitral valve regurgitation. Selective coronarography showed no significant stenosis on the LCCA. The coronary artery of the RCA is visualized after the administration of a contrast fluid into the LCCA. This documents an adequate supply of the RCA coronary arteries with LCCA and formation of extensive and large collateral vessels from the left coronary artery (Figure 1). There was no progression of findings compared to the examination five years ago. CT coronarography confirmed the findings of ARCAPA without another CHD. The RCA was shown as a strong dominant vessel with a diameter of 3 mm arising from the pulmonary artery (Figure 2). Due to the severe symptomatic aortic stenosis, surgery was recommended.

Cardiopulmonary bypass was standardly established by cannulation of the ascending aorta and the right atrium. The vent was inserted into the left ventricle via the right superior pulmonary vein. Visualization of the RCA in the pulmonary artery was absolutely necessary in order to apply the cardioplegic solution. Despite the fact that the ECHO did not confirm the dysfunction of the ventricle of the heart, the participation of the RCA origin congenital malformation in the patient’s symptomatology could not be excluded. Therefore, it was decided to transfer the RCA origin to the aortic wall. After administering the cardioplegic solution to the aortic root, the cardioplegic solution was also applied to the RCA through the incised PA. The RCA was explanted from the PA and translocated to the right coronary sinus of the aorta. Because of a defect after RCA button preparation, the PA root was closed with a pericardium patch (Figure 1). The stenotic aortic valve was explanted, and a No. 23 biological valve prosthesis was implanted to the aortic valve position. The aortotomy was then sutured. Cardiopulmonary bypass (CPB) time was 166 min and X-clamp lasted 132 min. The postoperative course was complicated by superficial wound infection treated with intravenous antibiotics and conservative therapy. The patient was discharged on the 12th postoperative day.

We report herein a case proving that even a pathological distance of one coronary artery can be asymptomatic if the pathophysiology of the coronary circulation is satisfactory.

## 3. Discussion

Prior to birth, a patent ductus arteriosus determines the equivalent pressure and oxygen saturation in the aorta and the PA. On the first day of life, together with the closure of ductus arteriosus, this results in a decrease in oxygen content, pressure, and perfusion of the anomalous coronary artery [15]. The absence of collateral circulation between the right and left coronary artery lead to a very early manifestation of symptoms of dilated cardiomyopathy and heart failure, with the incidence of sudden death in 90% of cases [3,8,9,15]. It is caused by decrease of pulmonary arterial pressure and coronary vascular reserve decrease [16]. This manifestation occurs typically at 2–3 months of age [7]. When collateral circulation develops extensively, left ventricular function can be preserved. This can lead to asymptomatic survival into adulthood [3]. On the other hand, ARCAPA is most commonly an asymptomatic disease, typically diagnosed as an incidental finding in patients from birth to over 90 years old [12,17]. The vascular resistance of the collateralized coronary circulation can lead to a balanced state with minimal shunting from the aorta to the truncus pulmonalis. On the other hand, this balance can be disturbed by changing the pressure ratios during exercise [16]. One-third to two-thirds of patients with ARCAPA are asymptomatic and have the best prognosis among all forms of anomalous arising of the coronary artery from the PA [4,14]. Manifestations of ARCAPA include dyspnea, fatigue, congestive heart failure, myocardial infarction, and sudden cardiac death [12]. In young patients, ARCAPA can be presented with exertional syncope, myocardial infarction, exercise-induced arrhythmias, or cardiac arrest [2,17]. Ohashi et al. presented a case report of a patient with the first sign of ARCAPA manifested by cardiac arrest during a marathon run [17]. It should be mentioned that only two case reports of cardiac arrest caused by ARCAPA appear in the literature [17]. Initial presentation of ARCAPA associated with rapid atrial fibrillation has also been reported [13]. The incidence of ARCAPA manifestation has two peaks, one shortly after birth and another in their forties and fifties [1,2]. Manifestation in childhood is caused by the coronary steal phenomenon; nevertheless, the typical manifestation in adults is often caused by atherosclerosis of the left coronary artery [1].

Before 1965, all coronary artery malformations were diagnosed during surgery or at autopsy only [1]. Electrocardiography (ECG) may show signs of ischemia, Q waves, and ST changes in the anterior and lateral leads, especially during exercise testing [6]. Imaging methods for an ALCAPA and ARCAPA-suspected patient include transthoracic echocardiography (TTE), computed tomography (CT) and magnetic resonance imaging (MRI). In comparison to ALCAPA, ARCAPA is easier to find in ECHO, and the main findings in adult patients include intraseptal coronary collaterals in using color flow Doppler mapping, dilatation of coronary arteries, increased flow in LCCA and flow between the RCA and the PA [1,17]. ECHO can raise the suspicion of a coronary anomaly in 40% of cases [2]. ARCAPA can be mistaken for a coronary fistula when there is a dilated LCCA and continuous or diastolic retrograde flow from an anomalous vessel to the PA. Another differential diagnosis of ARCAPA is right coronary artery atresia, also presented with intercoronary collaterals and dilated LCCA [2]. Concomitant incidence of ARCAPA and aortopulmonary window is presented with no, or less collateral circulation and tortuosity of coronary arteries in the ECHO image [1]. Cardiac angiography is the gold standard for ARCAPA diagnostic, with imaging of a complete map of the coronary vasculature [1]. The electrocardiogram can be either normal or can indicate left ventricular hypertrophy or deep Q waves in inferior leads, especially during exercise [2].

According to the 2020 ESC guidelines, surgical intervention due to ARCAPA is needed when symptomatology is presented or ventricular dysfunction or myocardial ischemia is presented as a consequence of the malformation [18]. A non-dominant RCA in the terrain of ARCAPA will generally not lead to clinically significant myocardial ischemia [15]. However, the guideline is based on a low level of scientific evidence, and the literature shows that surgery is not performed in only 7.8% of patients [13,14]. The literature shows that because ARCAPA can lead to increased risk of myocardial infarction and sudden cardiac death even in asymptomatic patients, surgical correction is recommended when ischemia is confirmed [1,2,17]. Surgical correction is associated with 2–3% perioperative mortality and longer life spans in comparison with patients without surgery [1]. Correction of ARCAPA can be performed through re-implantation to the aorta, through simple ligation or through ligation and aortocoronary bypass [12]. Simple ligation is associated with high early and late mortality and should be supplemented with an aortocoronary bypass [1]. Reimplantation is the most recommended surgery for this malformation. However, direct reimplantation is feasible since the proximal portion of the right coronary artery is long enough without significant branches [17]. This surgical procedure is feasible without cardiopulmonary bypass, and long-term patency is excellent, especially in the cohort of young patients [17].

## 4. Conclusions

A patient diagnosed with ARCAPA should be carefully examined for signs of ischemia. Scintigraphy with pharmacological and exercise imaging stress should be investigated. If signs of ischemia are positive, early surgical intervention can reverse the risk of sudden cardiac death. Current guidelines do not strictly express the indication of ARCAPA correction, in terrain when a patient undergoes another cardiac surgery. However, if we leave out the possibility of applying retrograde cardioplegia, visualization of the RCA orifice is necessary for antegrade cardioplegic solution application. In addition, if the ARCAPA is left and later atherosclerotic changes of the LCCA will progress, the patient is in higher risk of ischemic heart disease development. According to the authors, ARCAPA orifice transferee should be considered during any other cardiac surgery requiring cardiopulmonary bypass and cardiac arrest. The most appropriate surgical intervention is re-implantation of the right coronary artery into the aorta. If reimplantation is not possible, ligation of the ARCAPA origin from PA and aortocoronary bypass appear to be more appropriate therapy.

## Data Availability

No other data and documents are shared with the processed case report.

## References

[1] Ajam A., Rahnamoun Z., Sahebjam M., Sattartabar B., Razminia Y., Ahmadi Tafti S.H., Hosseini K. (2022). Cardiac imaging findings in anomalous origin of the coronary arteries from the pulmonary artery; narrative review of the literature. Echo Res. Pract..

[2] Mahmoud H., Cinteza E., Voicu C., Margarint I., Rotaru I., Aria A., Youssef T., Nicolescu A. (2022). Challenging Diagnosis of Anomalous Origin of the Right Coronary Artery from the Pulmonary Artery. Diagnostics.

[3] Rao S., Pathmanathan A., Khan A., Malik M., Chaudhuri D., Ford T., Byrum C., Smith F. (2022). Heart Failure as the Initial Presentation of Anomalous Left Coronary Artery From the Pulmonary Artery. J. Investig. Med. High. Impact Case Rep..

[4] Mishra A. (2021). Surgical management of anomalous origin of coronary artery from pulmonary artery. Indian J. Thorac. Cardiovasc. Surg..

[5] Cavalcanti L.R.P., Sa M., Escorel Neto A.C., Salerno P.R., Lima R.C. (2021). Anomalous origin of the left coronary artery from the pulmonary artery (ALCAPA) in adults: Collateral circulation does not preclude direct reimplantation. J. Card. Surg..

[6] Prandi F.R., Zaidi A.N., LaRocca G., Hadley M., Riasat M., Anastasius M.O., Moreno P.R., Sharma S., Kini A., Murthy R. (2022). Sudden Cardiac Arrest in an Adult with Anomalous Origin of the Left Coronary Artery from the Pulmonary Artery (ALCAPA): Case Report. Int. J. Environ. Res. Public Health.

[7] Ojha V., Pandey N.N., Kumar S., Ramakrishnan S., Jagia P. (2021). Anomalous origin of left main coronary artery from pulmonary artery: Patient characteristics and imaging associations on multidetector computed tomography angiography. J. Card. Surg..

[8] Alsamman M., Tuna K., Dunn S., Rifai F., Reed J. (2021). Adult Anomalous Left Coronary Artery Arising From the Pulmonary Artery (ALCAPA) Syndrome as First Presentation With Atrial Fibrillation in a Marathon Runner. Cureus.

[9] Wang X., Xia X., Huang W., Li X., Liu Y. (2022). Anomalous origin of the left coronary artery from the pulmonary artery as a rare cause of mitral valve prolapse: A case report. BMC Cardiovasc. Disord..

[10] Furuta A., Matsumura G., Shinkawa T., Niinami H. (2021). Long-term surgical results of anomalous origin of the left coronary artery from the pulmonary artery repair in infants and older patients. J. Card. Surg..

[11] Shibagaki K., Shiiku C., Kamiya H., Kikuchi Y. (2021). Anomalous Origin of the Left Anterior Descending Coronary Artery in an Adult. Thorac. Cardiovasc. Surg. Rep..

[12] Irvine D.S., Rozava K., Theodotou A., Evans R., Huang J. (2022). Anesthesia Consideration for a Patient With Incidentally Diagnosed Anomalous Origin of Right Coronary Artery Originating From Pulmonary Trunk (ARCAPA): A Case Study. Cureus.

[13] Sorgaard M.H., Kofoed K.F., Abdulla J. (2023). Anomalous origin of the right coronary artery from pulmonary artery in an adult presenting with rapid atrial fibrillation: A case report. Eur. Heart J. Case Rep..

[14] Scordino D., Venturi G., Nudi F., Tomai F. (2022). Multi-imaging evaluation and long-term outcome of a patient with chest pain and an anomalous right coronary artery arising from pulmonary artery: A case report. Eur. Heart J. Case Rep..

[15] Talebian Yazdi M., Robbers-Visser D., van der Bilt I.A.C., Boekholdt S.M., Koolbergen D.R., Planken R.N., Groenink M. (2022). Anomalous coronary artery from the pulmonary artery diagnosed in adulthood: A case series on variations of coronary anatomy and the diagnostic value of cardiac magnetic resonance imaging. Eur. Heart J. Case Rep..

[16] Achim A., Johnson N.P., Liblik K., Burckhardt A., Krivoshei L., Leibundgut G. (2023). Coronary steal: How many thieves are out there?. Eur. Heart J..

[17] Ohashi K., Itagaki R., Mukaida T., Miyazaki K., Ohashi K., Kawada M., Abe D. (2022). Cardiac Arrest in a 33-year-old Marathon Runner with Anomalous Right Coronary Artery Originating from the Pulmonary Artery. Intern. Med..

[18] Baumgartner H., De Backer J. (2020). The ESC Clinical Practice Guidelines for the Management of Adult Congenital Heart Disease 2020. Eur. Heart J..

